# Temporomandibular Joint Ankylosis in a Girl Child: Immunochemical Evaluation of Tissue Material Obtained from Repeated Arthroplasty Surgeries

**DOI:** 10.3390/dj11010016

**Published:** 2023-01-03

**Authors:** Nityanand Jain, Mara Pilmane, Andrejs Skagers, Shivani Jain, Pavlo Fedirko

**Affiliations:** 1 Department of Morphology, Institute of Anatomy and Anthropology, Riga Stradiņš University, Dzirciema Street 16, LV-1007 Riga, Latvia; 2 Department of Oral and Maxillofacial Surgery, Faculty of Dentistry, Riga Stradiņš University, Dzirciema Street 16, LV-1007 Riga, Latvia; 3 Department of Oral and Maxillofacial Surgery and Oral Implantology, Genesis Institute of Dental Sciences and Research, Ferozepur 152002, Punjab, India; 4 Institute of Radiation Hygiene and Epidemiology, State Institution-National Research Center for Radiation Medicine of the National Academy of Medical Sciences of Ukraine, Illenka Street 53, 04050 Kyiv, Ukraine

**Keywords:** TMJ, temporomandibular joint, ankylosis, immunohistochemistry, tissue remodeling, growth factors, cytokines

## Abstract

Temporomandibular joint ankylosis (TMJA) is a rare, but debilitating, condition that leads to TMJ joint hypomobility. Surgery is the mainstay for treatment, which is accompanied by rehabilitative and psychological support. Despite the advances in surgical techniques, the recurrence of TMJA post-surgery has been reported as a common complication. Therefore, it becomes essential to investigate and understand the histo-morpho-pathological processes governing these ankylotic changes. Given the lack of such studies in the literature, herein we present a case of a girl child who underwent primary surgery at the age of six years, followed by a second surgery at the age of twelve years. Ankylotic tissue samples collected during both surgeries were studied using various immunohistochemical markers for tissue remodeling, inflammation, antimicrobial activity, and transcriptional regulation. The expression of MMP-2 and -9 was downregulated in repeated surgery materials, whilst MMP-13 was rarely detected in both tissues. Strong MMP-8, TIMP-2, and TIMP-4 expressions were noted in both tissues, showing their anti-inflammatory and protective roles. Moderately strong expression of bFGF, FGFR-1, IL-1α, and TNF-α could indicate sustained tissue growth in the background of inflammation (wound healing). Interestingly, the expression of β-defensin-2 was found to be constant in both tissues, thereby indicating possible ECM remodeling and collagen breakdown. Finally, a moderate expression of RUNX-2, coupled with a low expression of WNT-1 and -3a, could indicate a slow and delayed bone regeneration process. Our results showcase the complex myriad of pathways that could be involved in the progression of TMJA and post-surgery healing processes. Immunopathological studies could aid in improving the diagnosis, treatment, and prognosis for patients affected with TMJA.

## 1. Introduction

The temporomandibular joint (TMJ) is a bilateral diarthrodial joint that is part of the stomatognathic system, an interrelated and interdependent anatomic unit involved in speech, reception, mastication, and deglutition of food [1]. Its dysfunction often leads to joint hypo- or hyper-mobility, thereby affecting the quality of life for the affected patients. Although multiple factors have been described to cause TMJ dysfunction, ankylosis of the TMJ joint presents a rare and arduous challenge for the patient and surgeon alike [2]. Ankylosis of the joint occurs due to the intracapsular bony or fibrous fusion of the disc condyle complex to the glenoid fossa, causing joint restriction and hypo-mobility [1,2]. It is characterized as a true joint pathology since the normal articulation is replaced by either bony mass or fibrous tissue [3].

The etiology of temporomandibular joint ankylosis (TMJA) can be either primary (pathogenesis affects the joint directly), as in case of infections or rheumatoid conditions, or secondary, as in the case of trauma and TMJ surgeries [1,2]. Though it can affect both children and adults, the condition is more commonly characterized in younger individuals [4]. Diagnosis of TMJA requires a mix of clinical (symptoms, history) and radiological investigations (CT, orthopantomograms). However, in most pediatric cases, TMJA often goes undiagnosed for years until there is severe facial and functional impairment [5]. Neglect and ignorance on the part of parents, especially after trauma, along with poor patient management at hospitals at the time of injury, are pivotal in the missed early diagnosis of TMJA progression [6].

Apart from early detection and proper patient management, the provisioning of rehabilitation and psychological support to the patient is equally important in deciding the success rates [5]. The utility of such multi-specialty management of the TMJA patient is clear from the declining incidence of TMJA and decrease in associated complications in the developed countries in the global west [1]. Regardless of the age of detection, surgery is the mainstay of patient management—gap arthroplasty, inter-positional arthroplasty, and joint reconstruction or total joint replacement. In younger patients, reconstruction with autogenous costochondral graft (CCG) has been advocated, due to the probable growth potential of the graft tissue [2,7,8]. Nonetheless, recurrence due to continued ankylotic changes post-surgery remain a frequently reported complication [9].

Given the complex pathoetiology and difficulties in the management and healing process of TMJA, it becomes essential to study the histological changes in the ankylotic tissue microenvironment that governs these pathological changes. However, the limited availability of tissue material, the rarity of the condition itself, and late diagnosis and surgical intervention (usually when patient has long-standing or end-stage disease) has restricted the possibilities to conduct extensive research in human subjects. In the literature, only a handful of human tissue-based histopathological studies have been reported [10,11,12,13,14]. In these studies, apart from general morphological appearance, processes related to neoangiogenesis [10], collagenization [11], cellular stress [12], bone resorption [13], and tissue growth [14] have been described immunohistochemically. Hence, in our present case report study, we furthered our understanding of various related processes by immunohistochemical investigation of the processes related to tissue remodeling, growth, inflammation, antimicrobial activity, and transcriptional regulation in two separate tissue samples obtained from the same patient six years apart.

## 2. Case Presentation

A 6-year-old healthy girl child presented to our department with a severely restricted mouth opening due to a unilateral left-sided, bony, complete intra-articular TMJA between the lower jaw, zygoma, and temporal bone. History was significant for a mandibular condylar fracture without dislocation on the same side, which was not treated or managed conservatively by the parents. The presentation and history of the patient was clear and indicative of a traumatic etiology. Localized imaging was performed in accordance with the established clinical practices. For this patient, primary arthroplasty of the TMJ was planned. During the arthroplasty, the child underwent rib autotransplantation—3 cm of fifth rib bone was harvested and capped by 6 mm of costochondral cartilage—and ankylotic tissue was collected for immunohistochemical evaluation. The tissue material collected displayed bone tissue, along with fragments of fibrous cartilage tissue.

The patient recovered well without any complications. No complaints were reported during the first three years of the surgery. However, in the next years, the patient noticed a progressive reduction of the oral cavity opening. At the time of re-presentation, after six years from primary surgery, the oral cavity opening was ≤ 2–3 mm between the incisors, thereby confirming re-ankylosis. Surgery was performed again, the ankylotic bony blocks were removed, and arthroplasty was performed. The surgery went without any complications, and the follow-up for the patient was uneventful. Patient tissue material (bony and fibrous cartilage tissue) was also collected at the time of second surgery for examination. The collected material was archived and evaluated at the Institute of Anatomy and Anthropology, Riga Stradinš University.

## 3. Immunohistochemical Analysis

The resected tissues from both surgeries were fixed in Stefanini’s solution and decalcified using “Decalcifer, rapid” (I.T. Baker company, catalogue no. ITB RS 155800054, The Netherlands) solution for 24 h. The tissues were then dehydrated through a graded series of ethanol, embedded in paraffin, and sectioned into 5 μm thin slices. For routine morphological evaluation, staining with hematoxylin and eosin (H&E) was performed. Following this, routine biotin-streptavidin immunohistochemistry (IHC) reactions were performed using primary antibodies against various proteins of interest (Table 1) [15].

To evaluate the IHC reaction, a previously developed semiquantitative scale was used: “0”—no immunoreactive cells found in the visual field; “+”—few immunoreactive cells are seen in the visual field; “++”—moderate number of immunoreactive structures are seen in the visual field; “+++”—numerous immunoreactive structures are seen in the visual field; and “++++”—abundant immunoreactive structures are seen in the visual field [16].

## 4. Immunohistochemistry Results

**Figure 1:**
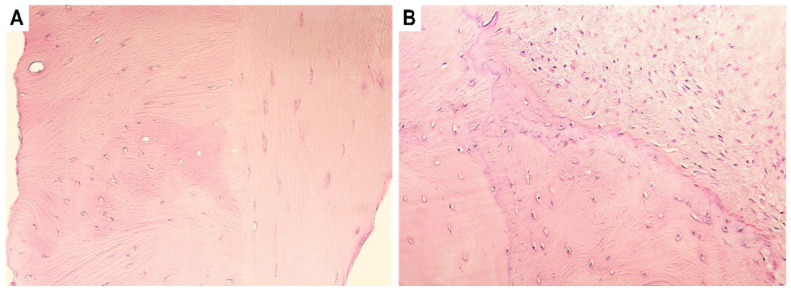
Routinely stained hematoxylin and eosin slides (H&E). (**A**) Note the chaotic organization of the ankylotic bone lamellae in the tissue obtained during primary surgery. (**B**) Non-uniform bone development surrounded by fibrotic tissue can be seen in the tissue obtained during the repeated surgery six years later. Original magnification, 250×.

Routinely stained slides revealed a chaotic arrangement of the bone collagen fibres (lamellae) and non-uniform bone deposits on the border, along with the presence of fibrotic soft tissue both during the primary and the repeated surgery (Figure 1). Additionally, there were no visually recognizable defects seen in the fibrous cartilaginous fragments.

### 4.1. Evaluation of Expression of Tissue Remodelling Factors

**Figure 2:**
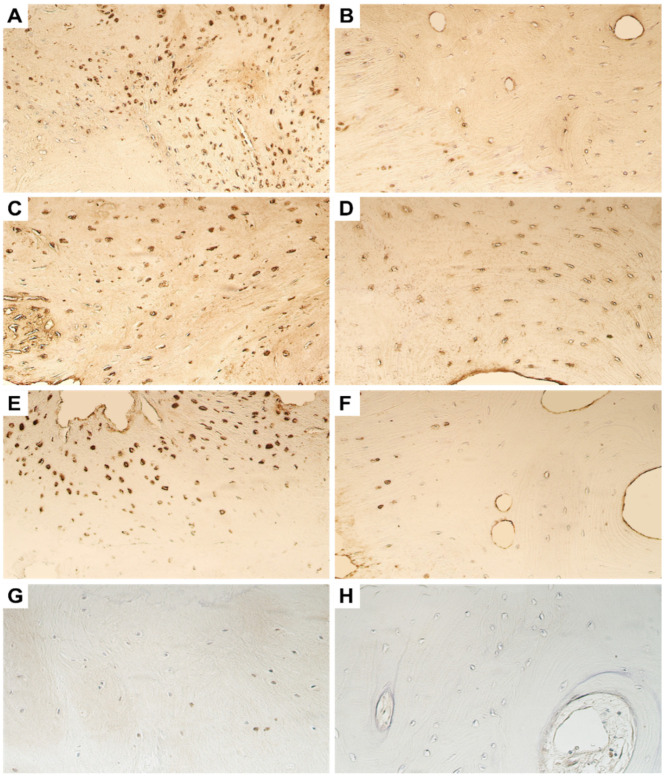
Microphotographs of the ankylotic bone and fibrous cartilage immunostained by MMP-2, -8, -9, and -13. (**A**) Numerous positive bone osteocytes for MMP-2 can be seen in the primary surgery tissue. (**B**) Note the presence of only few MMP-2-positive osteocytes and chondrocytes in the repeated surgery tissue. (**C**) Numerous MMP-8-positive osteocytes can be seen in the primary surgery tissue. (**D**) Numerous MMP-8 immunopositive osteocytes can also be seen in the repeated surgery tissue. (**E**) Moderate MMP-9-positive osteocytes with a patchy distribution can be seen in the primary surgery tissue. (**F**) In the repeated surgery tissue, only few MMP-9-positive osteocytes and chondrocytes can be visualized. (**G**) Scantly distributed MMP-13-positive osteocytes can be noted in the primary surgery tissue material. (**H**) There is complete absence of MMP-13-positive cells in the repeated surgery tissue. Original magnification, 250×.

Whilst we saw numerous MMP-2-positive bone cells in the bone and fibrous cartilage tissue obtained during the primary surgery, only few MMP-2-positive osteocytes and chondrocytes could be visualized in the tissue collected during the repeated surgery (Figure 2A,B). MMP-8 was found to be the most strongly and widely expressed metalloproteinase in the ankylotic tissue. Numerous MMP-8-positive osteocytes and chondrocytes were seen in both the primary and repeated surgery materials (Figure 2C,D).

The patchy distribution of moderate number of MMP-9-positive cells was seen in the entire tissue material from the primary surgery in the girl, whilst the repeated surgery material showed just a few MMP-9-immunopositive cells (Figure 2E,F and Table 2). MMP-13 was scarcely visualized in the ankylotic tissue material. Only a few positive cells were seen in the bony tissue and fibrous cartilage in the primary surgery material, whilst none to few cells were seen in the repeated surgery material (Figure 2G,H).

**Figure 3:**
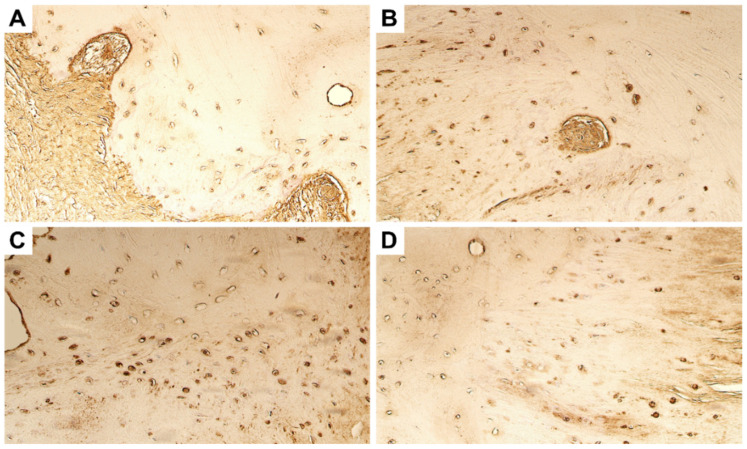
Microphotographs of the ankylotic bone and fibrous cartilage immunohistochemically stained by TIMP-2 and TIMP-4. Numerous TIMP-2-positive osteocytes and chondrocytes in the (**A**) primary surgery tissue material and (**B**) repeated surgery material. Original magnification, 250×. Moderate to numerous TIMP-4-positive cells could be visualized in the tissue obtained during the (**C**) primary surgery and (**D**) repeated surgery. Original magnification, 250×.

Whilst the expression levels of positive inducers of tissue remodeling were variable, the inhibitors of metalloproteinases showed a more stable expression. Numerous immunoreactive cells were visualized for TIMP-2 and TIMP-4 in the bony tissue material, both in the primary and repeated surgery tissue (Figure 3). In the fibrous cartilage tissue, TIMP-2 expression was universally found to be stronger than that of TIMP-4 (Table 2).

### 4.2. Evaluation of Expression of Growth Factors and Cytokines

**Figure 4:**
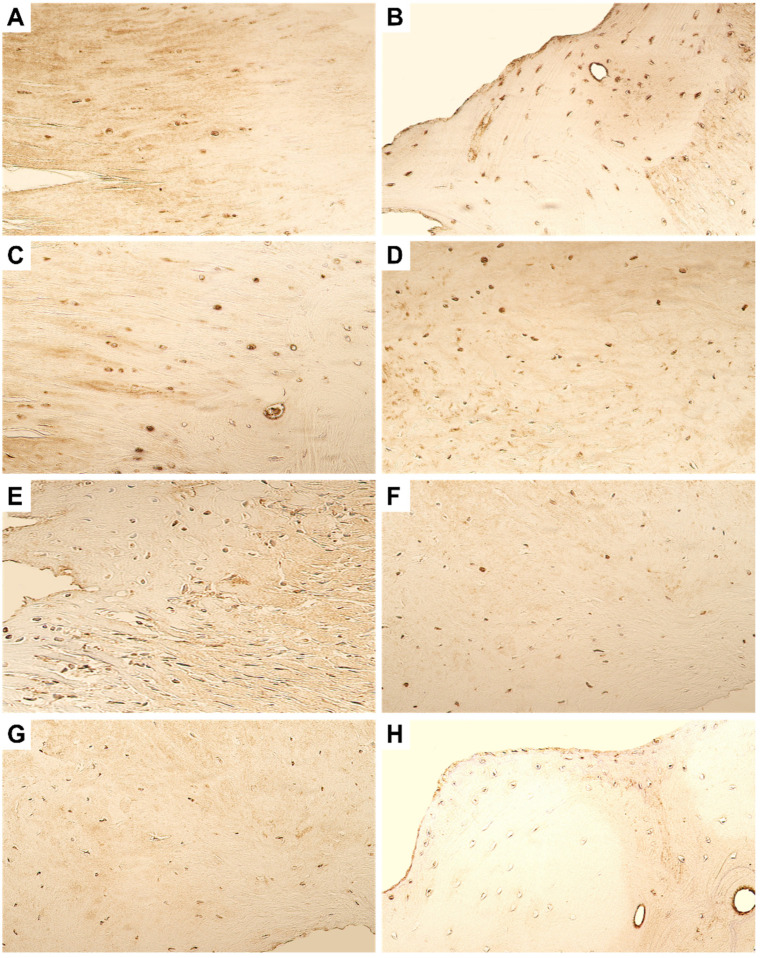
Microphotographs of the ankylotic bone and fibrous cartilage tissue immunostained for bFGF, FGFR-1, IL-1α, and TNF-α. (**A**) Few bFGF-positive chondrocytes can be seen in the primary surgery tissue material. (**B**) Moderate bFGF-positive osteocytes can be noted in the repeated surgery tissue material. (**C**) Moderate FGFR-1-positive chondrocytes in the fibrous cartilage of the primary surgery tissue material can be seen. (**D**) In the repeated surgery material, moderate FRFR-1-positive cells can be visualized. (**E**) Moderate IL-1α-positive cells can be seen in the primary surgery material. (**F**) Moderate IL-1α-positive cells can also be noted in the repeated surgery material. (**G**) Numerous-positive TNF-α cells in the primary surgery tissue. (**H**) Note presence of few TNF-α-positive osteocytes in the repeated surgery material. Original magnification, 250×.

Immunostaining with bFGF revealed only a few positive cells in the fibrous cartilage of the primary surgery tissue. However, the bony tissue was visualized with moderate bFGF-positive cells in both primary and repeated surgery tissue material (Figure 4A,B). Expression of FGFR-1, on the other hand, was found to be stable and uniform in both bony and fibrous cartilaginous tissue (Table 2). No difference in expression was noted in primary and repeated surgery material (Figure 4C,D).

In regard to the cytokines, the expression of IL-1 was also found to be stable with moderate number of osteocytes and chondrocytes positive in both primary and repeated surgery material (Figure 4E,F). The expression of TNF-α was found to be stronger in the primary surgery material, with moderate to numerous cells showing immunopositivity. However, in the repeated surgery material, only few TNF-α-positive were visualized (Figure 4G,H).

### 4.3. Evaluation of Expression of Antimicrobial Peptide

**Figure 5:**
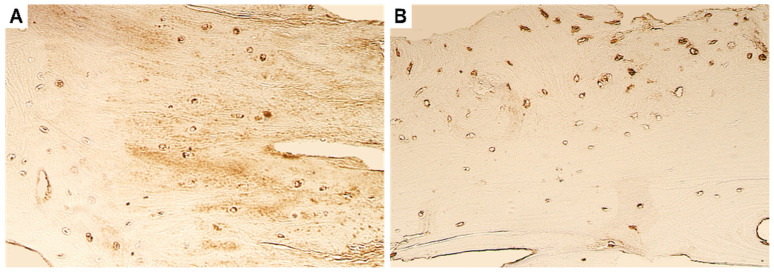
Microphotographs of the fibrous cartilage obtained during the (**A**) primary surgery and (**B**) repeated surgery. Note the presence of moderate number of BD-2-positive cells in the tissue material. Original magnification, 250×.

Expression of β-defensin-2 was found to be stable in both the primary and repeated surgery material. Both the tissues showed the presence of a moderate number of immunopositive osteocytes and chondrocytes (Figure 5).

### 4.4. Evaluation of Expression of Transcription Factors

**Figure 6:**
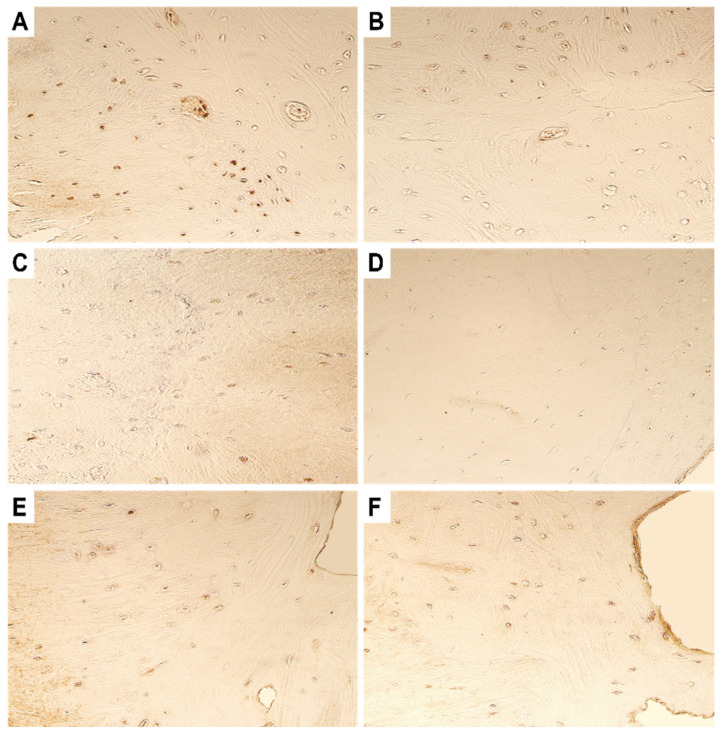
Microphotographs of the fibrous cartilage and ankylotic bone tissue immunostained with RUNX-2, WNT-1, and WNT-3a. (**A**) Moderate number of RUNX-2-positive chondrocytes and osteocytes can be noted in the primary surgery tissue. (**B**) In the repeated surgery tissue, only few RUNX-2-positive cells can be seen. (**C**) Few WNT-1 osteocytes can be visualized in the bony tissue from primary surgery. (**D**) No WNT-1-positive cells could be seen in the repeated surgery material. (**E**) Few WNT-3a-positive chondrocytes and osteocytes in the primary surgery material can be noted. (**F**) In the repeated surgery tissue, only few WNT-3a-positive osteocytes were seen. Original magnification, 250×.

Regarding the expression of the transcriptional factors and gene proteins, a moderate number of RUNX-2-positive cells were seen in the primary surgery material, whilst the material from the repeated surgery showed only few RUNX-2 immunopositive cells (Figure 6A,B). The expression of WNT-1 was scant in the ankylotic material. Only few WNT-1-positive osteocytes were seen in the primary surgery material, but none were observed in repeated surgery material (Figure 6C,D). The expression of WNT-3a protein did not show any differences in the primary and repeated surgery material and was visualized with the presence of few positive cells.

## 5. Discussion

In our present study, we present a case of a six-year-old girl child who suffered from TMJA and underwent primary surgical intervention, followed by a second surgical intervention at the age of twelve years, due to continued ankylotic changes. Immunohistochemical evaluation of the tissue material collected during both surgeries showed changes in the tissue architecture and microenvironment, in terms of processes governing tissue remodeling, inflammation, growth, antimicrobial defense, and transcriptional processes. Such changes can advance our understanding of the histopathological disturbances in the normal growth processes in the TMJ joint region.

Firstly, we examined the expression of tissue remodeling factors metalloproteinases and their inhibitors. MMPs are a family of 23 zinc-containing, calcium-dependent endopeptidases, which can degrade and remodel extracellular matrix (ECM) proteins [17]. Based on their sub-cellular distribution and specificity for components, MMPs can be categorized into different sub-families [17]. MMP-2 and MMP-9 are gelatin-binding MMPs that are secreted into the ECM for the degradation of gelatins, collagens (I, IV, V, VII, X, XI), fibronectin, laminin, etc. Additionally, they are needed for angiogenesis, neurogenesis, and mediation of apoptotic processes. MMP-8 and MMP-13, on the other hand, are simple hemopexin domain-containing MMPs that are also secreted into the ECM for the degradation of collagens (II, III, VIII, X), aggrecan, and entactin. Their function is fundamental for the proper growth and development of bone and ligaments.

In the bone tissue, MMP-2 is overexpressed and promotes mineralization of the bone, whilst osteoclast-secreted MMP-9 is responsible for bone resorption and altering the bone strength [18,19]. However, in the later stages, MMP-9 has been postulated to be expressed by the inflammatory cells [18] and to prevent the accumulation of hypertrophic chondrocytes [20]. In knockout mouse models, a lack of MMP-9 has been associated with delayed bone vascularization, the ossification of hypertrophic cartilage, and the formation of more brittle bones [21,22]. Additionally, it has been shown that CD34+ endotheliocytes are present along the cartilage–bone junction and accelerate the calcification and formation of bony TMJA [10]. Another study also reported the presence of aberrant cartilage that was characterized by hypertrophic chondrocyte-like cells at the bone/cartilage interface [13]. In our study, we found that the expression of both MMP-2 and MMP-9 was strong in the primary surgery tissue but was reduced in the repeated surgery material. Increased expression in the primary surgery tissue could indicate their role in the promotion of the ossification of the TMJ and joint cartilage, whilst a reduced expression in the repeated surgery tissue could be indicative of acceleration of the collagenization process in the TMJ. In both cases, there seems to be a disbalance in the expression patterns of both these MMPs.

In fact, collagenization of the residual joint space and articular surface has been described previously, which could lead to osteoarthritis and enthesopathy under prolonged and continued mechanical stress [11]. Both collagen type-I and -II have been implicated in promoting these changes in TMJA patients. Given these changes, it is quite interesting to see the nil or low-level expression of MMP-13 in the tissue materials. MMP-13 is a key MMP expressed by hypertrophic chondrocytes, periosteal cells, and osteoblasts in the cartilage tissue [23], which handles the breakdown of cartilage in osteoarthritic joints [24]. The only MMP to demonstrate a stable expression was MMP-8, which is secreted by activated neutrophils (hence independent of dysregulations in the bone and cartilage metabolism) in the presence of pro-inflammatory signals. It seems to play a rather protective role, since the sustained expression of MMP-8 aids in the reduction of severity of arthritis, reduced inflammation, and bone erosion [25].

Next, we examined the expression of tissue inhibitors of MMPs or TIMPs, a family of four endogenous protein regulators that are present in the ECM. They are crucial for regulating the levels of MMPs, and they exert their influence on the cell phenotype, inflammation, and growth factors [17]. An imbalance in the MMP-2: TIMP-2 ratio (in favor of TIMP-2) has been linked with excessive accumulation of the ECM in the joints [26]. Interestingly, whilst, on one hand, TIMP-2 inhibits MMP-2, it is also responsible for the activation of MMP-2, which then leads to fibrotic changes due to tissue inflammation [27]. TIMP-4 remains an under-investigated member of the TIMP family but has been postulated to play a protective role by inhibiting fibrotic changes and inflammation [28]. We found that, whilst in the primary surgery material the MMP-2: TIMP-2 ratio remained stable, it favored TIMP-2 in the repeated surgery tissue. Perhaps given the increased levels of inflammation, both TIMP-2 and -4 were expressed in higher levels.

Regarding the expression of the growth factors bFGF and FGFR-1, bFGF has been shown to stimulate angiogenesis and promote the wound-healing process. Furthermore, a lack of bFGF could induce the development of osteoarthritis [29]. bFGF also stimulates glycosaminoglycans (GAGs) synthesis and promotes collagen synthesis [30]. However, a concentration-dependent role of bFGF have been postulated, whereby a lower concentration promotes biosynthesis, and a higher concentration promotes cellular proliferation [30]. Since it was not possible for us to benchmark our bFGF levels to normal tissue or to quantify the expression levels, we cannot, at this stage, be certain of the exact function that bFGF might promote in TMJA tissue. Microarray analyses have revealed that FGFR-1 is expressed in the periosteum of the condyle and fossa in a time- and region-dependent manner [31]. Mouse models have revealed that the inactivation of the FGFR-1 pathway leads to slowing of the TMJ osteoarthritis progression, due to the promotion of autophagic activity [32]. Furthermore, a deficiency of FGFR-1 was found to decrease MMP-13 expression [32]. We suspect that sustained bFGF levels could have affected the levels of FGFR-1 in both tissues, thereby promoting osteoarthritic changes in the tissue.

In healthy TMJ tissue, there is absence of pro-inflammatory cytokines, such as IL-1α and TNF-α. However, excessive loading or use of the joint leads to the beginning of a cascade of tissue alteration processes, of which IL-1 is the most dominant and first to be released [33,34]. IL-1 usually leads to the depletion of ECM structural proteins, such as collagen and blocking of its expression confers cartilage and bone protective effects [35]. Similarly, TNF-α leads to bone resorption (via osteoclastic activation) and promotes local tissue inflammation. Together with IL-1α, TNF-α can form resorption pits during an inflammatory process [36]. We found a sustained moderate expression of IL-1α in tissue materials from both surgeries; however, the expression of TNF-α was drastically reduced. We could postulate that the decrease in TNF-α could show a slow-down in the bone resorption process during the second surgery. Concerning β-defensin-2 (BD-2), we found a sustained expression in both tissue materials. BD-2 is expressed due to either the presence of microbes or pro-inflammatory signals in the epithelial and mucosal tissue. However, earlier findings of BDs in the TMJ synovial fluid have led to the emergence of their role in promoting the breakdown of the ECM and articular cartilage [37]. In fact, BDs were absent in healthy TMJ articular cartilage, but were found in osteoarthritic TMJ cartilage without septic changes [38]. Therefore, sustained expression of BD-2 in our materials could indicate their destructive role in the pathogenesis of TMJA.

Finally, we investigated the possible role of three transcriptional factors in the pathogenesis of TMJA-RUNX-2 and WNT-1 and -3a. RUNX-2 has been shown to be crucial for the maturation of hypertrophic chondrocytes embryonically; however, its deletion postnatally can also cause tissue disorganization and an associated reduction in production of cartilage matrix [39]. The low expression of RUNX-2, like FGFR-1, can lead to decreased MMP-13 expression [39]. Hence, we assume that, in repeated surgery material, the lower levels of RUNX-2 and MMP-13 could show the promotion of cartilage growth and bone remodeling. WNTs are secreted glycoproteins that regulate fracture healing, bone mass, and bone regeneration in postnatal life. Both WNT-1 and -3a have been found to be significantly upregulated 6 months post TMJA surgery [40]. However, our findings of lower WNTs expression could indicate a delay in bone regeneration, something which is commonly seen in TMJA patients. Functional stimulation has been tried for inducing spontaneous bone regeneration in young adults [41].

Nonetheless, we want to highlight that the findings in the present study could not be potentially generalized for all patients. Differences in the etiopathogenesis of TMJA could lead to differences in the tissue expression of these proteins and factors. We also acknowledge that more factors and pathways need to be investigated in the future to understand the complete picture. Similarly, environmental factors and personal habits, such as chewing techniques, the overuse of the joints, etc., can affect our result findings. For example, certain rheumatic and autoimmune processes could also be present as underlying mechanisms. Furthermore, since the patient had high TNF-α during the primary surgery, we could have prescribed anti-TNF-α therapy. However, given that the case was performed about 12 years ago, such therapeutic modalities were still not widely prescribed. The post-surgery rehabilitation process and patient compliance are equally important factors that need to be considered, whilst explaining the differences in the tissue expression of the investigated proteins. However, with the present study, we highlight the role of various pathways in the promotion and dysregulation of wound healing and tissue growth in pediatric patients.

## 6. Conclusions

Variances in the expression of MMP-2 and -9 showed a flip in their function from promoting ossification to accelerated collagenization (sustained by low MMP-13 levels). The sustained, strong expression of MMP-8 could play a protective role by reducing joint inflammation and bone erosion. bFGF and FGFR-1 both promote osteoarthritic changes in the tissue, whilst IL-1α and TNF-α are most likely associated with pro-inflammatory mediation and resorptive changes. Whilst RUNX-2 could promote cartilage growth and bone remodeling, the low expression of WNTs indicates slow bone regeneration.

## Figures and Tables

**Table 1:** Description of the primary antibodies used for evaluation.

Primary Antibody	Full Name	Function	Dilution	Manufacturer
MMP-2	Matrix metallopeptidase 2	Tissue remodeling	1:400	Biorbyt Limited (Cambridge, UK)
MMP-8	Matrix metallopeptidase 8	Tissue remodeling	1:100	Biorbyt Limited (Cambridge, UK)
MMP-9	Matrix metallopeptidase 9	Tissue remodeling	1:100	Biorbyt Limited (Cambridge, UK)
MMP-13	Matrix metallopeptidase 13	Tissue remodeling	1:100	Santa Cruz (Dallas, TX, USA)
TIMP-2	TIMP metallopeptidase inhibitor 2	Tissue remodeling	1:50	Santa Cruz (Dallas, TX, USA)
TIMP-4	TIMP metallopeptidase inhibitor 4	Tissue remodeling	1:100	Abcam (Cambridge, UK)
bFGF	Basic fibroblast growth factor (FGF2)	Growth factor	1:200	Abcam (Cambridge, UK)
FGFR-1	Fibroblast growth factor receptor 1	Growth factor	1:100	Biorbyt Limited (Cambridge, UK)
IL-1α	Interleukin-1α	Cytokine	1:100	Biorbyt Limited (Cambridge, UK)
TNF-α	Tumor necrosis factor alpha	Cytokine	1:200	Abcam (Cambridge, UK)
ΒD-2	Beta defensin-2	Antimicrobial peptide	1:100	Santa Cruz (Dallas, TX, USA)
RUNX-2	RUNX family transcription factor 2	Transcription factor	1:250	Abcam (Cambridge, UK)
WNT-1	Wnt family member 1	Transcription factor	1:100	Abcam (Cambridge, UK)
WNT-3a	Wnt family member 3a	Transcription factor	1:800	Abcam (Cambridge, UK)

**Table 2:** Immunohistochemical (semi-quantitative) grading of various tissue factors visualized in the ankylotic fibrous cartilage and bone tissue.

Tissue Material	MMP-2	MMP-8	MMP-9	MMP-13	TIMP-2	TIMP-4	bFGF	FGFR-1	IL-1α	TNF-α	BD-2	RUNX-2	WNT-1	WNT-3a
Primary surgery tissue														
Bone	+++	+++	++	+	+++	+++	++	++	++	+++	++	++	+	+
Fibrous cartilage	+++	+++	++	+	+++	++	+	++	++	++	++	++	0	+
Repeated surgery tissue														
Bone	+	+++	+	0	+++	+++	++	++	++	+	++	+	+	+
Fibrous cartilage	+	+++	+	0	+++	++	++	++	++	+	++	+	0	+

Note: “0”—no immunoreactive cells found in the visual field; “+”—few immunoreactive cells are seen in the visual field; “++”—moderate number of immunoreactive structures are seen in the visual field and “+++”—numerous immunoreactive structures are seen in the visual field.

## Data Availability

Not applicable.
